# Right Heart Morphology of Candidate Patients for Transcatheter Tricuspid Valve Interventions

**DOI:** 10.1007/s13239-021-00595-y

**Published:** 2021-12-01

**Authors:** Omar K. Khalique, Vladimir Jelnin, Andreas Hueske, Mathew Lawlor, Martin B. Leon, Susheel K. Kodali, Deniz Akkoc, Eriq Pettway, Rebecca T. Hahn, Nadira B. Hamid, Isaac George, Amisha Patel, Vivian Ng, Torsten P. Vahl, Tamim M. Nazif, Andrew W. Siefert

**Affiliations:** 1 Structural Heart and Valve Center, Columbia University Medical Center, 5-501C, 177 Fort Washington Avenue, New York, NY 10032 USA; 2 Hackensack University Structural and Congenital Heart Center, 30 Prospect Avenue, 5 Main, Room 5640, Hackensack, NJ 07601 USA; 3 Cardiac Implants LLC, 25 Lake Terrace, Tarrytown, NY USA

**Keywords:** Heart, Tricuspid valve, Tricuspid regurgitation, Anatomy, Morphology, Computed tomography, Medical device, Design, Transcatheter therapies, Interventional cardiology, Medical imaging, Modeling

## Abstract

**Purpose:**

This study quantitatively evaluated the phasic right heart morphology of candidate patients for a transcatheter tricuspid valve intervention (N=32) and of subjects with trace to no tricuspid regurgitation (*N* = 14).

**Methods:**

Cardiac computed tomography angiography (CCTA) and transthoracic/transesophageal echocardiography (TTE/TEE) images were analyzed using dedicated research and clinical software. Using CCTA, the phasic right atrial and ventricular volumes, annulus dimensions, annulus-to-right coronary artery (RCA) distances, circumferential topography of the annular tissue shelf, vena cava dimensions (inferior and superior), vena cava positions, axis angles, and annular excursions were quantified. Using TTE/TEE, leaflet geometry, regurgitation, hemodynamics, and heart function were quantified. Measurements within and between groups were quantitatively compared with regression analyses to explore relationships between right heart features.

**Results:**

The phasic position and orientation of the vena cava and the circumferential topography of the annular tissue shelf were quantitatively presented for the first time. The candidate patient group exhibited greater chamber dimensions, enlarged vena cava, distended vena cava positions, positional shallowing of the annular tissue shelf, geometric annular distortion, leaflet distention, moderate or greater regurgitation, and impaired ventricular function. Atrial volume correlated strongly with directional vena cava positions as well as with annular dimensions. Annulus-to-RCA distances and annular excursions were comparable between groups.

**Conclusions:**

This study provides new and further insight to the right heart morphology and functional characteristics of candidate patients for a transcatheter tricuspid valve intervention. These data provide a platform from which these patients can continue to be better understood for further improving transcatheter system design and use.

**Supplementary Information:**

The online version contains supplementary material available at 10.1007/s13239-021-00595-y.

## Introduction

Functional tricuspid regurgitation (FTR) is the most prevalent tricuspid valve (TV) disease.^24^ This disease is characterized by progressive right atrial and ventricular enlargement, tricuspid annulus dilatation, leaflet tethering, and incomplete leaflet closure. FTR is often diagnosed at a late stage and with comorbidities, making many patients ineligible to undergo an open-heart surgery. The long-term outcomes for these unmet patients are poor, even when treated with optimal medical therapy.^10,19^

Less invasive transcatheter therapies have been proposed to treat these patients. These systems are designed to advance through the body’s venous vasculature, to maneuver within the beating heart, and to deploy implant(s) to the heart’s tissues. Currently proposed and developed systems have included edge-to-edge leaflet repair devices, TV replacement valves, heterotopic caval valve implants, leaflet spacers, annuloplasty systems, and devices to reshape the right heart chamber dimensions.^2,17,26^

A large influence on the design of the device delivery systems, implants, patient-implant sizing, and procedures is the phasic morphology of the right heart. Recent research has quantified phasic right heart chamber dimensions and volumes, tricuspid annulus dimensions, annulus-to-right coronary artery (RCA) distances, and inferior vena cava (IVC) dimensions in FTR patients.^7,8,11,15,16,20,21,27^ These data however have been unable to capture other critical features for transcatheter therapies, including the position of the vena cava with respect to the TV, vena cava delivery axis angles and heights, the topography of the tissue encircling the TV annulus, annulus excursion, and their correlation to other right heart features.^3^

These knowledge and data gaps are attributed to several factors. Echocardiography is the cardiac imaging modality of choice but restrictions in right heart imaging windows limit the measurements that can be recorded.^4,22^ Computed tomography and magnetic resonance imaging can quantify these missing features but require time-intensive manual approaches significantly limiting their routine quantification.^2,6^ A more comprehensive knowledge of these right heart morphological features, their variation, and complexities exhibits both possibility and promise for aiding transcatheter system design, sizing, and device-procedure development.

This study quantitatively evaluated the phasic right heart morphology of transcatheter patient candidates (*N* = 32). Cardiac computed tomography angiography (CCTA), transthoracic echocardiography (TTE), and transesophageal echocardiography (TEE) images were analyzed using dedicated research and clinical software. The phasic right atrial and ventricular volumes, annulus dimensions, annulus-to-RCA distances, topography of the annular tissue shelf, dimensions of the vena cava (inferior and superior), vena cava positions, axis angles, annular excursions, tricuspid regurgitation, leaflet geometry, hemodynamics, and heart function were quantified. As many measures are being reported for the first time, all measurements were repeated in subjects with trace or no regurgitation (*N* = 14) to explore the impact of right heart disease and range of values that may be seen clinically.

## Materials and Methods

### Patient Selection

A retrospective study on de-identified medical imaging data was conducted. Forty-six patients were selected and analyzed as a part of an Institutional Review Board-approved protocol with waiver of informed consent (Columbia University Medical Center, New York City, NY). The studied groups included thirty-two candidate patients for a transcatheter TV intervention with moderate or greater FTR (≥ 3 + TR group) and fourteen patients with trace to no TR (≤ 1 + TR group). The inclusion criteria for all patients consisted of no previous TV procedure, the availability of TTE and TEE imaging, and a multi-phase CCTA scan. Further inclusion criteria for the medical images included no major imaging artifacts and adequate right sided CCTA contrast for morphological measurements.

### Echocardiography Data and Analysis

Transthoracic and transesophageal echocardiography images were analyzed as a means to provide clinical context to the studied patient groups and to provide measures of tricuspid leaflet geometry, tricuspid regurgitation, and cardiac function to supplement the phasic CCTA-based morphological measures. Comprehensive echocardiography imaging was available for all patients and analyzed using syngo (Siemens, Erlangen, Germany) and QLab (Philips, Amsterdam, Netherlands).

Measures of right ventricular (RV) size and function were performed according to the American Society of Echocardiography guidelines,^12^ including RV basal and mid-dimensions, RV length, fractional area change, and RV systolic tissue Doppler velocity. The 4-chamber tenting height and tenting area of the leaflets were also determined. TR grading was performed using multiple methods according to guidelines and quantitative Doppler methods.^28^ RV outflow tract and TV stroke volumes were determined from cross-sectional areas and velocity time integrals. For the left heart, multiple measurements of regurgitation severity, atrial volume index, and ejection fraction were performed according to guidelines.^12^

### Cardiac Computed Tomography Angiography Data and Analysis

All patients previously underwent an electrocardiographically gated CCTA using a 320 detector-row system (Toshiba Medical Systems, Otawara, Japan). A contrast-enhanced tri-phase protocol was followed for the ≥ 3 + TR group.^23^ A transcatheter aortic valve planning protocol was followed for the ≤ 1 + TR group.^9^ All scans were reconstructed with a slice thickness of 0.5 mm in 5% increments across one full R-R electrocardiogram interval (20 phases of the cardiac cycle). Per the patient inclusion criteria, all scans exhibited sufficient right sided contrast for the morphological measurements.

**Figure 1:**
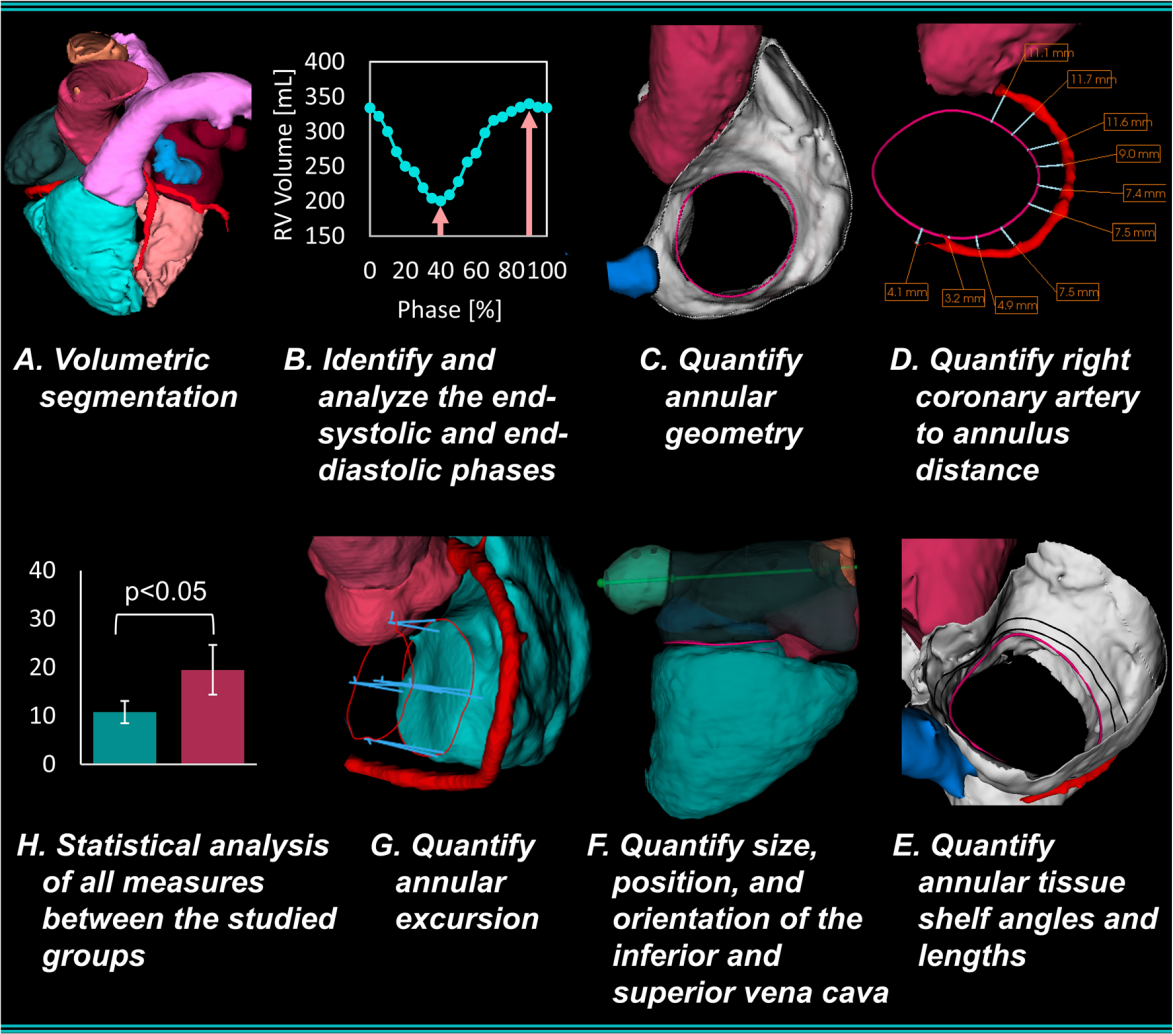
Cardiac computed tomography angiography analysis overview.

CCTA data was analyzed using CI Vision (LARALAB GmbH, Munich, Germany, Cardiac Implants LLC, Tarrytown, NY). This research software provides a highly automized workflow for CCTA data processing and analysis. This software was utilized over commercially available software to permit a more comprehensive quantification of the right heart features for which other software do not include within their workflows or alternatively require prohibitive, time-intensive manual approaches. This software was used to quantify the phasic right heart morphology as follows:

Fully automatic volumetric reconstructions of the heart for each phase of the R-R electrocardiogram interval were completed (Fig. 1A). The end-systolic and end-diastolic phases were identified by determining the phases with minimum and maximum RV volumes (Fig. 1B). The phases in which end-systole and end-diastole occurred were recorded along with the corresponding RA and RV volumes.

**Figure 2:**
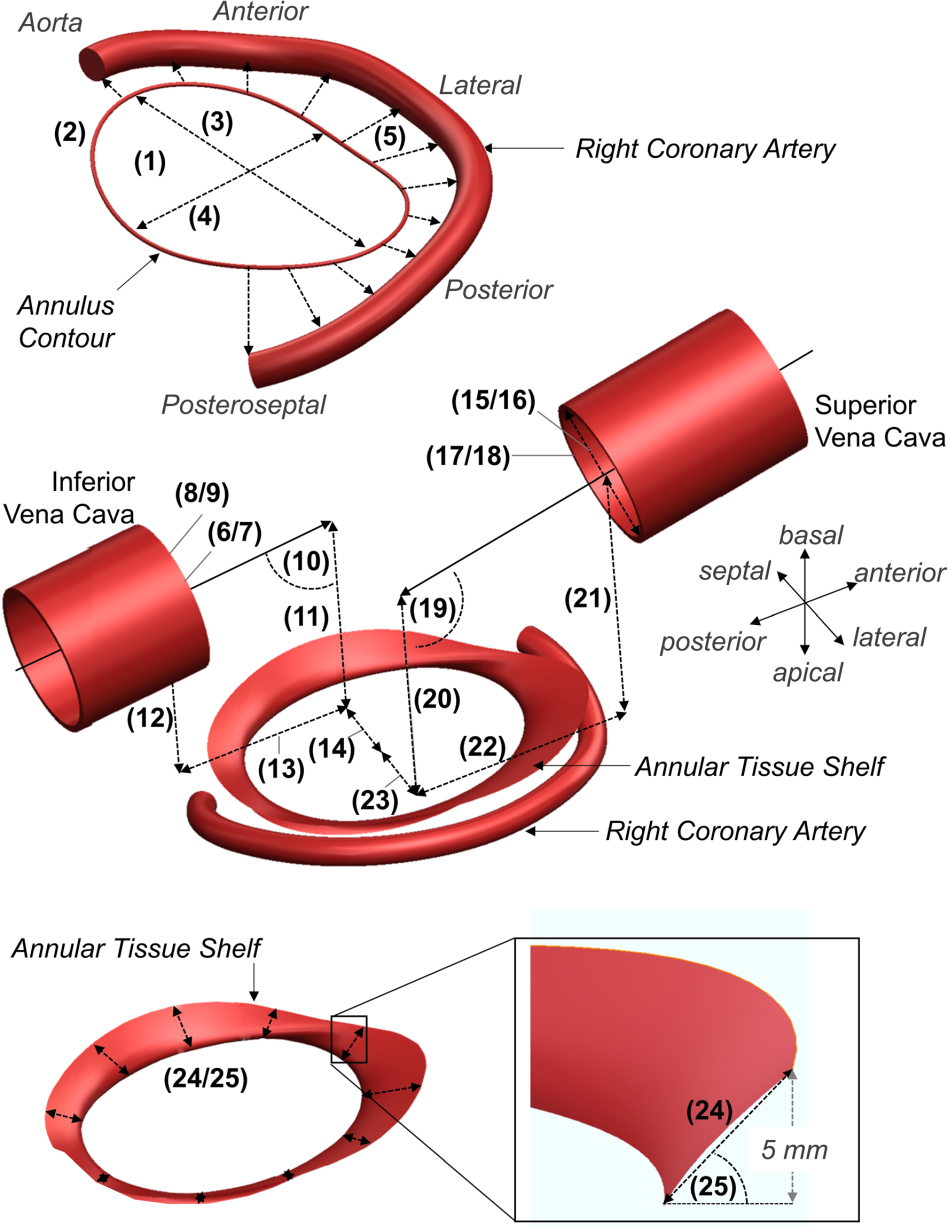
Cardiac computed tomography angiography measurement overview: (1) annular area, (2) 3D Perimeter, (3) maximum diameter, (4) minimum diameter, (5) annulus-to-RCA distance, (6/7) maximum and minimum inferior vena cava (IVC) diameters, (8/9) IVC area and Perimeter, (10) IVC axis angle, (11) IVC axis height above the septolateral annular axis, (12) Basal distance from annulus center to IVC, (13) Posterior distance from annulus center to IVC, (14) Septal distance from annulus center to IVC, (15/16) maximum and minimum superior vena cava (SVC) diameters, (17/18) SVC area and perimeter, (19) SVC axis angle, (20) SVC axis height above the septolateral annular axis, (21) Basal distance from annulus center to SVC, (22) Anterior distance from annulus center to SVC, (23) Lateral distance from annulus center to SVC, (24) tissue shelf length, (25) tissue shelf angle.

For both phases, a semi-automatic segmentation of the tricuspid annulus at the leaflet hinge was completed with the annular area, 3D perimeter, maximum diameter, minimum diameter, and eccentricity (minimum/maximum diameter) automatically determined (Figs. 1C and 2). Using the defined contour of the tricuspid annulus, twelve absolute distances from the annulus to the adjacent segmented wall of the RCA were automatically determined (Figs. 1D and 2).

The topology of the tissue shelf encircling the TV was then automatically quantified at ten rotationally symmetric positions around the segmented annulus (Figs. 1E and 2). To standardize the length and angle measurements across patients and phases, each measure was based on a best-fit plane through the segmented annulus and a 5-mm atrial height to the segmented annular contour (Fig. 2). This height was chosen anecdotally, as most annular based devices implant to or expand into this tissue region. The tissue between the annular plane and the 5-mm atrial height was defined as the annular tissue shelf (Fig. 2).

The maximum diameter, minimum diameter, area, and perimeter of the inferior vena cava (IVC) and superior vena cava (SVC) at the cavoatrial junction were then quantified (Figs. 1F and 2). The axis of delivery through the IVC and SVC centerlines were then determined from the visible length of each vessel within the scan. Using these axes, their relative angle and height atrial to the septolateral axis of the tricuspid annulus was determined (Fig. 2). The relative positions of the IVC and SVC openings to the center of the valve annulus were then quantified as pictured in Fig. 2. Using the phasic three-dimensional positions of the vena cava with respect to the annulus center, the total and directional excursions of the annulus centroid were calculated (Fig. 1G).

### Inter-Software and User Variability

CI Vision is a research software not yet available for commercial use. The precision and agreement of select measurements by CI Vision were therefore compared to commercially available software packages that included Materialise Mimics (Materialise, Leuven, Belgium), 3Mensio (3Mensio Medical Imaging, Bilthoven, Netherlands), and HeartNavigator (Philips, Best, Netherlands). Annular area, perimeter, maximum diameter, minimum diameter, and the basal distance of the SVC from the TV annulus were determined in two phases of the R-R electrocardiogram interval (40 %, 80 %) from thirty-eight patients by expert users and compared using Bland-Altman reporting the mean bias and 95% limits of agreement. Measurements from each software and metric were also assessed using correlation, reporting the Pearson correlation coefficient (*r*), 95 % confidence interval, and *p* values, respectively.

### Statistical Analysis

All statistical analyses were completed using Minitab (Minitab LLC, State College, PA). Normality for continuous variables were assessed by the Anderson-Darling test and expressed as a mean ± one standard deviation (SD) and range. Intergroup comparisons for parametric data were assessed using two sample *t* tests, and non-parametric data was assessed with Mann–Whitney *U* test. Intragroup comparisons were completed using a paired-samples *t* test and Wilcoxon signed-rank test for non-parametric variables. Categorical variables were assessed using Pearson’s Chi-squared tests and Fisher’s Exact tests. Pearson correlations between measurements were completed reporting the correlation coefficient (*r*), 95% confidence interval (CI), and *p* values, respectively. Multiple regression was used to evaluate relationships between anatomical features and annulus-to-RCA distances as well as the morphology of the annular tissue shelf.

## Results

### Group and Echocardiography Characteristics

There was no difference between the ≤ 1 + TR and ≥ 3 + TR groups for age (78.8 ± 6.0 years vs. 78.8 ± 9.9 years, *p* = 0.519), body surface area (1.8 ± 0.3 m^2^ vs. 1.8 ± 0.2 m^2^, *p* = 0.542), and sex (females, *N* = 10 (71%) vs. *N* = 18 (56%), *p* = 0.332). Subjects within the ≥ 3 + TR group exhibited a transvalvular pacemaker lead (*N* = 3), mitral annuloplasty ring (*N* = 2), and mitral replacement (*N* = 6). One subject of the ≤ 1 + TR group had a prior mitral replacement. The echocardiography measurements from the studied patient groups are tabulated in Table 1. The ≥ 3 + TR group expectedly exhibited greater TV leaflet distention, tricuspid regurgitation, enlarged right heart measurements, and impaired RV function. Left heart metrics were comparable between the groups with exception to a reduced left ventricular ejection fraction within the ≥ 3 + TR group.

**Table 1:** Echocardiography characteristics (mean ± SD) with the following abbreviations: TV-tricuspid valve, and EROA-effective regurgitant orifice area.

Metric	≤ 1 + TR	≥ 3 + TR	*p* value
Tricuspid regurgitation severity (*n*)			
None	1	0	0.304
Trace	13	0	<0.0005
Moderate	0	5	0.303
Severe	0	23	<0.0005
Torrential	0	4	0.298
TV leaflet tenting height (cm)	0.5 ± 0.2	1.0 ± 0.3	<0.0005
TV leaflet tenting area (cm^2^)	0.8 ± 0.3	2.4 ± 1.8	0.001
Quantitative doppler EROA (cm^2^)	–	1.5 ± 1.0	–
Tricuspid valve stroke volume (mL)	–	165 ± 44	–
Quantitative regurgitation volume (mL)	–	98 ± 47	–
Right ventricular outflow tract stroke volume (mL)	66 ± 14	59 ± 22	0.131
RV end-diastolic diameter (base) (cm)	4.2 ± 2.3	5.4 ± 0.9	<0.0005
RV end-diastolic diameter (mid) (cm)	2.9 ± 1.3	3.9 ± 0.9	<0.0005
RV length (cm)	6.3 ± 0.8	7.5 ± 0.9	<0.0005
RV end-diastolic area (cm^2^)	13.1 ± 4.8	27.9 ± 8.1	<0.0005
RV end-systolic area (cm^2^)	7.6 ± 3.7	18.0 ± 6.0	<0.0005
RV fractional area change (%)	44 ± 12	35 ± 11	0.054
Left atrial volume index (mL/m^2^)	69 ± 26	73 ± 32	0.916
Left ventricular ejection fraction (%)	65 ± 11	56 ± 10	0.022
Left ventricular outflow tract stroke volume (mL)	72 ± 21	62 ± 14	0.121
Mitral regurgitation severity (*n*)			
None	2	2	0.574
Trace	5	7	0.467
Mild	4	11	1.000
Moderate	2	11	0.286
Severe	1	1	0.521

### Timing of End-Systole and -Diastole with Chamber Volumes

**Figure 3:**
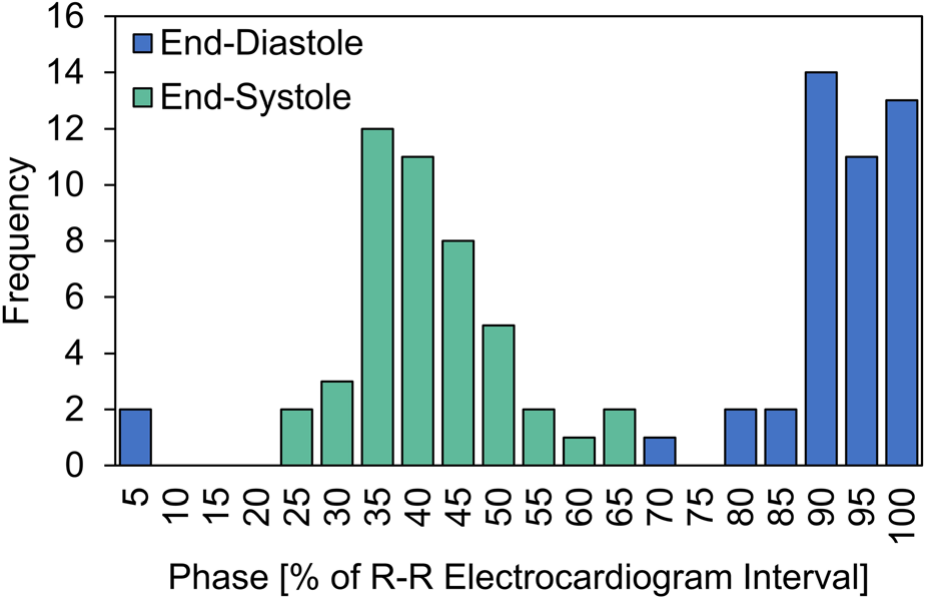
Histogram quantifying the frequency in which the end-systolic and end-diastolic phases occurred based on volumetric computed tomography measurements of minimum and maximum right ventricular volume.

End-systole was most frequently observed in the 35–40% phases whereas end-diastole most frequently occurred in the 90–100% phases (Figure 3). The CCTA-based measurements of RA and RV volume are reported in Table 2. The chamber volumes for the ≥ 3 + TR group were 2- to 3-fold greater than those of the ≤ 1 + TR group (*p* < 0.0005). The phasic RA and RV volumes were observed to exhibit only a moderate degree of correlation (*r* = 0.50, 95% CI 0.32–0.64, *p* < 0.0005).

**Table 2:** Right atrial (RA) and right ventricle (RV) measurements at end-systole (ES) and end-diastole expressed as a mean ± SD (minimum–maximum).

Measurement	≤ 1 + TR	≥ 3 + TR	*p* value
RA volume (mL)			
ES	122 ± 32 (81–182)	368 ± 215 (145–1016)	<0.0005
ED	87 ± 34 (46–148)	328 ± 205 (124–889)	<0.0005
RV volume (mL)			
ES	76 ± 30 (42–138)	155 ± 64 (64–349)	<0.0005
ED	139 ± 34 (89–200)	280 ± 100 (117–596)	<0.0005

### Tricuspid Annulus Dimensions

Tricuspid annulus measurements are summarized in Table 3. For both phases, the ≥ 3 + TR group expectedly exhibited larger annular areas and perimeters. Maximum and minimum diameters were 24–40% larger within the ≥ 3 + TR group (*p* < 0.0005). The ≥ 3 + TR group annuli were more circular but maintained a preserved degree of ovality. The annular dimensions were generally observed to reduce from end-diastole to end-systole, whose mean reductions were found to be similar between groups.

**Table 3:** Tricuspid annulus measurements at end-systole (ES) and end-diastole (ED) expressed as a mean ± SD (minimum–maximum) with *p* values for comparisons between the ≤ 1 + TR and ≥ 3 + TR groups as well as *p* values for comparisons between ES and ED (ES-ED).

Measurement	≤ 1 +TR	≥ 3 + TR	*p* value
Annular area (cm^2^)			
ES	10.7 ± 2.2 (8.4–16.0)	19.5 ± 5.1 (11.7–34.2)	<0.0005
ED	11.6 ± 2.1 (9.1–16.3)	20.9 ± 5.3 (12.7–36.7)	<0.0005
ES-ED *p* value	0.009	<0.0005	
Annular perimeter (mm)			
ES	119 ± 11 (105–144)	158 ± 20 (124–209)	<0.0005
ED	124 ± 10 (109–146)	163 ± 20 (127–217)	<0.0005
ES-ED *p* value	0.007	<0.0005	
Maximum diameter (mm)			
ES	41 ± 4 (36–49)	53 ± 7 (41–69)	<0.0005
ED	44 ± 4 (39–49)	55 ± 7 (45–74)	<0.0005
ES-ED *p* value	0.015	<0.0005	
Minimum diameter (mm)			
ES	33 ± 7 (25–41)	46 ± 6 (33–59)	<0.0005
ED	34 ± 4 (27–43)	48 ± 7 (36–68)	<0.0005
ES-ED *p* value	0.096	<0.0005	
Eccentricity (mm/mm)			
ES	0.78 ± 0.14 (0.53–0.95)	0.86 ± 0.06 (0.72–0.94)	0.058
ED	0.77 ± 0.09 (0.57–0.88)	0.88 ± 0.06 (0.73–0.96)	<0.0005
ES-ED *p* value	0.754	0.188	

**Figure 4:**
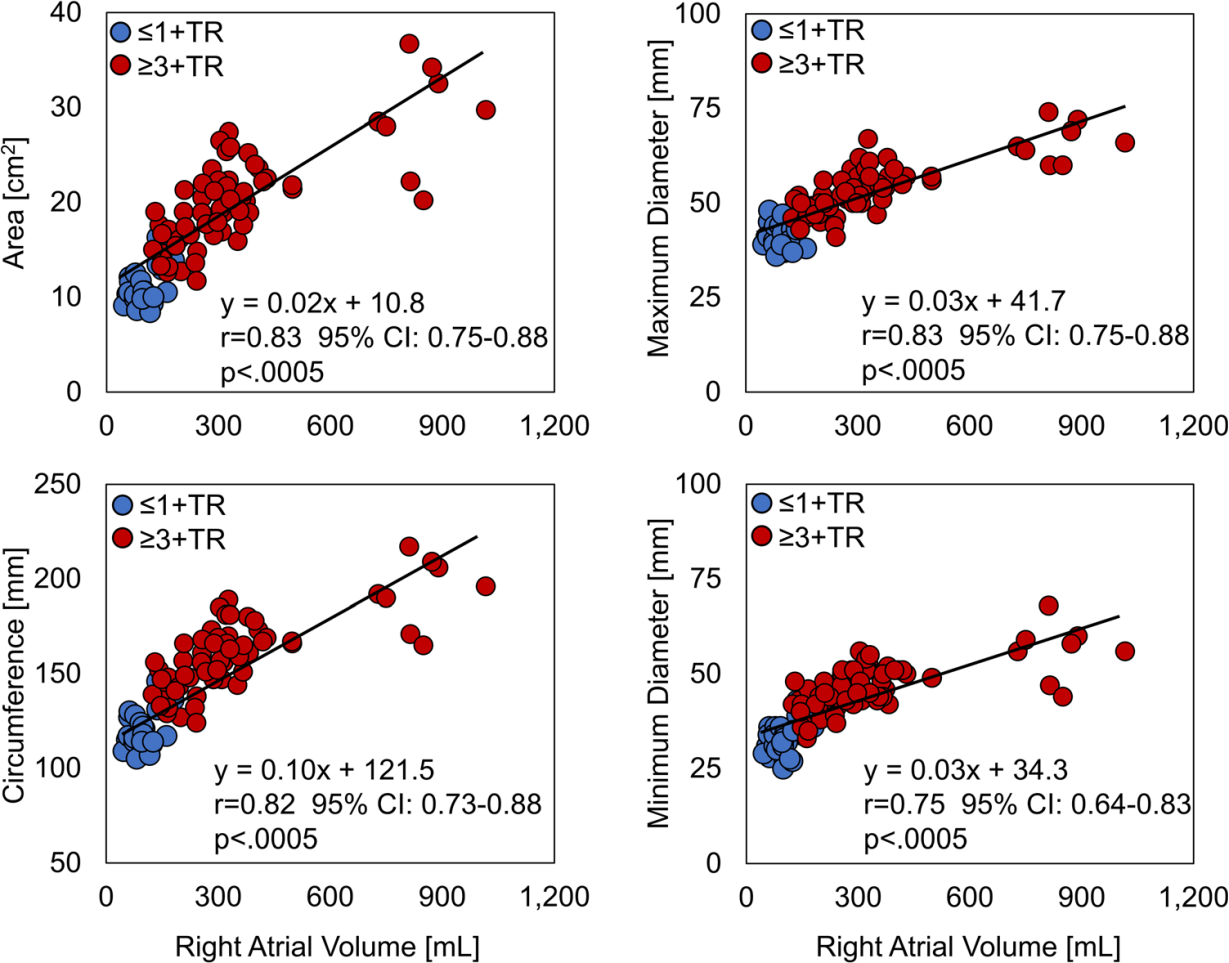
Correlations between right atrial volume and tricuspid annular dimensions (blue markers: ≤ 1 + TR group, red markers: ≥ 3 + TR group).

The tricuspid annular dimensions were observed to strongly correlate with RA volume (Figure 4). Some overlap between groups can be seen within the plots, with greater variance in annular dimensions for patients with RA volumes exceeding 700 mL. Based on linear regression, the maximum and minimum diameters were observed to increase at a similar rate with increasing RA volume. Annular dimensions were also observed to strongly correlate with RV volumes, although to a lesser extent, with Pearson r correlation coefficients in the range of 0.68–0.73.

### Right Coronary Artery

**Figure 5:**
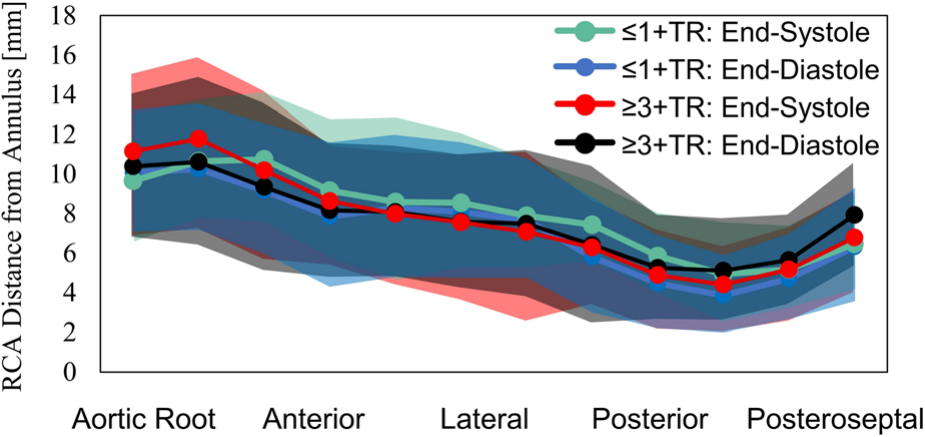
Mean distance of the RCA from the annulus with shaded regions representing 1-standard deviation.

For all patients and phases, the mean annulus-to-RCA distance was 7.9 ± 3.8 mm with a range of 0.5–23.2 mm (Figure 5). The mean annulus-to-RCA decreased in its distance along the annular perimeter, reaching a minimum near the posterior aspect before diving apically into the ventricle. No differences in annulus-to-RCA distance were observed between groups and phases. Using multiple regression, no correlations were observed between annulus-to-RCA measures and other right heart features.

### Annular Tissue Shelf

**Figure 6:**
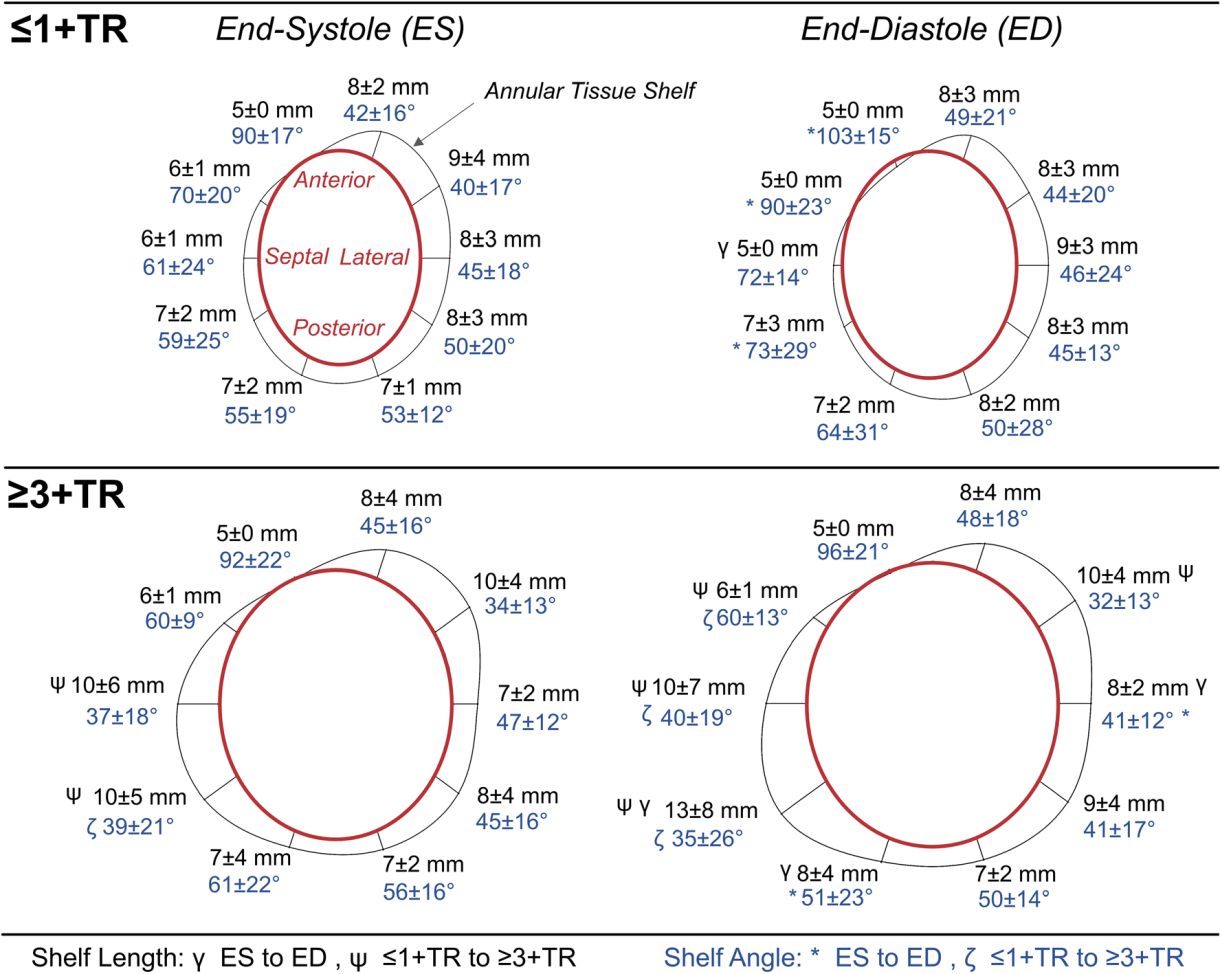
En-Face view of the mean annular tissue shelf lengths and angles (blue font) for the patient groups; symbols denote statistical differences at the *p* < 0.05 level.

The topography of the annular tissue shelf for the differing phases and patient groups is schematically represented in Figure 6. For the ≤ 1 + TR group, the mean tissue shelf angle and length were similar in magnitude from the anterior to posterolateral region in both end-systole and end-diastole. In both phases, the shelf angle increased proceeding clockwise from the posterior to septal region, where it became vertical near the shelf’s continuity with the aortic valve. The verticalization of the shelf at these positions was further increased at end-diastole (*p* < 0.05).

In the ≥ 3 + TR group, the coronary sinus was located more towards the valve plane, resulting in a wider shelf and shallower tissue angle in the posteroseptal region (Figure 6). Similar to the ≤ 1 + TR group, the shelf became vertical in the anteroseptal region at the aortic continuity. This influence of the aorta was only observed at one location in the ≥ 3 + TR group as opposed to two in the ≤ 1 + TR group. This was attributed to the enlarged dimensions of the ≥ 3 + TR group annulus, and the resulting spatial locations of the rotationally symmetric shelf measurements. When examining differences between the groups, differences in shelf length and angle existed mostly in the posteroseptal region. Using multiple regression, no relationships were observed between annular shelf measures and other right heart features.

### Inferior Vena Cava

The IVC size, axis, and position relative to the tricuspid annulus center is tabulated in Table 4. The maximum IVC diameter at the cavoatrial junction was enlarged within the ≥ 3 + TR group (IVC minimum diameter, area, and perimeter are presented in Supplemental Table 1). The delivery axis through the IVC centerline maintained an approximately parallel angle to the TV plane in both end-systole and end-diastole. Systolic increases in IVC axis height were observed. In both groups, the position of the IVC relative to the annulus was commonly biased towards the septal aspect of the annulus. All directional IVC distances were consistently greater in the ≥ 3 + TR group, with exception to the septal dimension at end-systole.

**Table 4:** Inferior vena cava (IVC) measurements at end-systole (ES) and end-diastole expressed as a mean ± SD (minimum–maximum) with *p* values for comparisons between the ≤ 1 + TR and ≥ 3 + TR groups as well as *p* values for comparisons between ES and ED (ES-ED), please see additional measures in Supplemental Table 1.

Measurement	≤ 1 + TR	≥ 3 + TR	*p* value
IVC maximum diameter (mm)			
ES	28 ± 5 (18–38)	34 ± 6 (25–45)	0.002
ED	29 ± 4 (22–38)	33 ± 6 (25–48)	0.006
ES-ED *p* value	0.869	0.206	
IVC axis angle (degrees)			
ES	85 ± 10 (62–98)	87 ± 11 (66–110)	0.517
ED	80 ± 11 (58–93)	85 ± 11 (65–107)	0.154
ES-ED *p* value	0.010	0.037	
IVC axis height (mm)			
ES	35 ± 8 (24–52)	51 ± 12 (34–86)	<0.0005
ED	27 ± 8 (16–47)	43 ± 12 (26–78)	<0.0005
ES-ED *p* value	<0.0005	<0.0005	
IVC position relative to the annulus center (mm)			
Basal			
ES	33 ± 9 (11–49)	49 ± 12 (29–76)	<0.005
ED	21 ± 10 (4–46)	39 ± 12 (21–72)	<0.005
ES-ED *p* value	<0.0005	<0.0005	
Posterior			
ES	25 ± 6 (18–38)	32 ± 10 (16–50)	0.009
ED	26 ± 6 (16–39)	34 ± 9 (18–58)	0.001
ES-ED *p* value	0.576	0.006	
Septal			
ES	15 ± 7 (7–31)	20 ± 9 (0–39)	0.084
ED	9 ± 7 (0–20)	16 ± 8 (0–36)	0.005
ES-ED *p* value	0.002	<0.0005	

**Figure 7:**
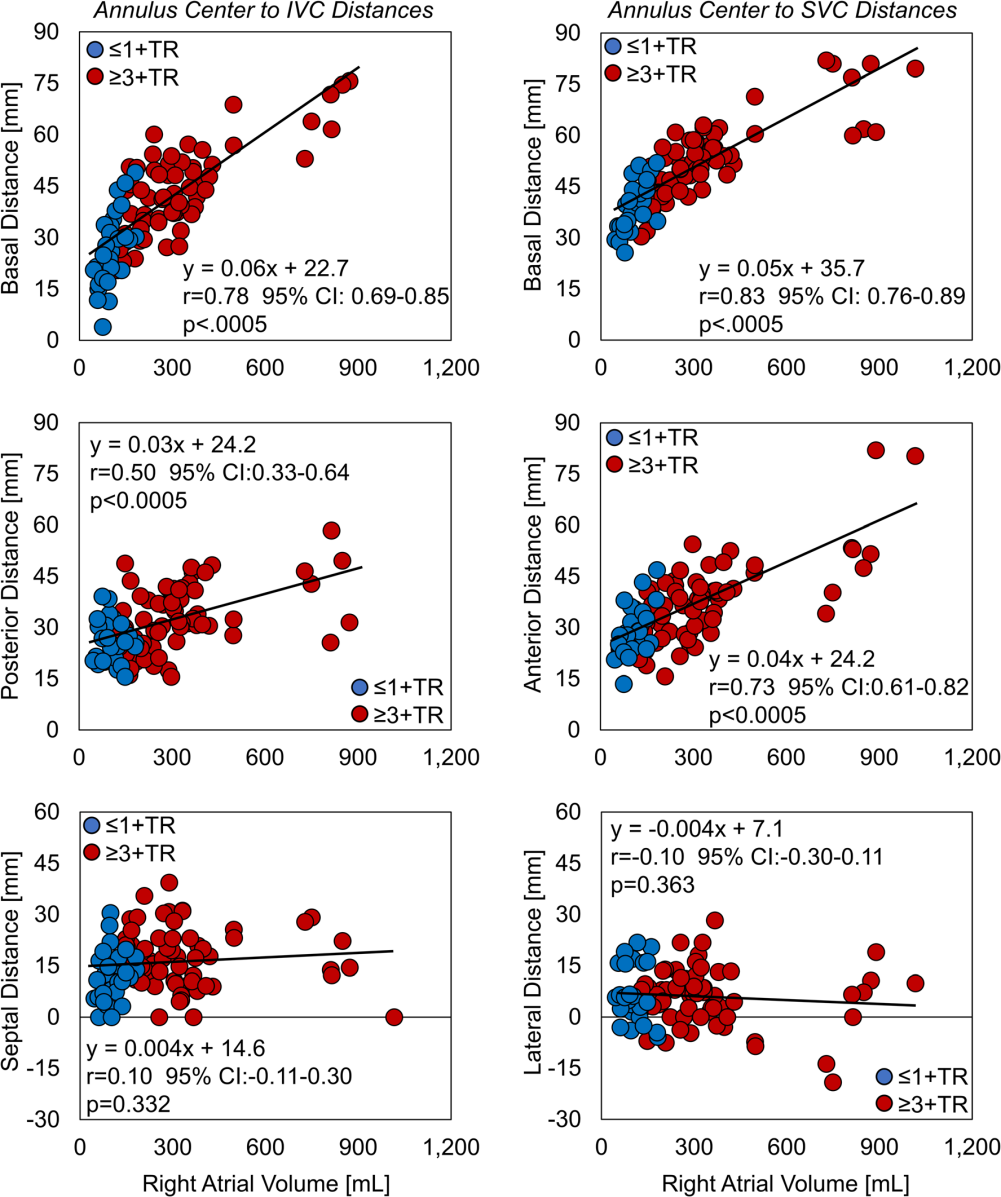
Correlations between right atrial volume and the directional distances from the annulus center to the inferior vena cava (IVC) (left column) and the directional distances from the annulus center to the superior vena cava (SVC) (blue markers: ≤ 1 + TR group, red markers: ≥ 3 + TR group) (Note: the IVC position was unable to be determined in one patient).

Of all measures, the RA volume was found to correlate most strongly with the directional positions of the IVC (Left Column of Figure 7). The RA volume exhibited strong correlations with the basal IVC distance, and lesser so with the posterior distance, with no correlation in the septal distance. Correlation was additionally used to assess any relationships between each of the IVC positional directions. Weak to no correlation was observed between each of the directional components (basal vs. septal: *r* = 0.27, 95% CI 0.07–0.45, *p* = 0.010; basal vs. posterior: *r* = 0.12, 95% CI − 0.09 to 0.32, *p* = 0.247; posterior vs. septal: *r* = 0.10, 95% CI − 0.12 to 0.30, *p* = 0.376).

### Superior Vena Cava

The SVC size, axis, and position relative to the tricuspid annulus center is reported in Table 5. The SVC diameter at the cavoatrial junction was significantly increased within the ≥ 3 + TR group (SVC minimum diameter, area, and perimeter are presented in Supplemental Table 2). The delivery axis angle was practically similar between groups and phases despite observing differences that were statistically significant. The SVC axis height was greater within the ≥ 3 + TR group whose height increased at end-systole. Patients within the ≥ 3 + TR group exhibited SVC positions that were more basal and anterior than those within the ≤ 1 + TR group. In both groups, the lateral positioning of the SVC varied greatly (Table 5, Right column of Figure 7).

**Table 5:** Superior vena cava (SVC) measurements at end-systole (ES) and end-diastole expressed as a mean ± SD (minimum–maximum) with *p* values for comparisons between the ≤ 1 + TR and ≥ 3 + TR groups as well as *p* values for comparisons between ES and ED (ES-ED), please see additional measures in Supplemental Table 2.

Measurement	≤ 1 + TR	≥ 3 + TR	*p* value
SVC maximum diameter [mm]			
ES	20 ± 3 (15–27)	29 ± 4 (22–39)	<0.0005
ED	18 ± 3 (13–24)	29 ± 5 (21–39)	<0.0005
ES-ED *p* value	<0.0005	0.185	
SVC delivery axis angle [degrees]			
ES	101 ± 4 (92–107)	100 ± 7 (81–111)	0.948
ED	110 ± 5 (101–120)	102 ± 7 (88–116)	<0.0005
ES-ED *p* value	<0.0005	0.015	
SVC delivery axis height [mm]			
ES	36 ± 6 (26–46)	50 ± 11 (33–78)	<0.0005
ED	26 ± 8 (16–37)	44 ± 11 (21–69)	<0.0005
ES-ED *p* value	<0.0005	<0.0005	
SVC position relative to the annulus center [mm]			
Basal			
ES	42 ± 6 (32–52)	56 ± 11 (38–81)	<0.0005
ED	35 ± 6 (26–47)	50 ± 11 (30–82)	<0.0005
ES-ED *p* value	0.001	<0.0005	
Anterior			
ES	32 ± 7 (24–47)	40 ± 11 (23–80)	0.009
ED	25 ± 6 (14–38)	37 ± 12 (16–82)	<0.0005
ES-ED *p* value	<0.0005	0.001	
Lateral			
ES	6 ± 9 (− 6 to 22)	6 ± 8 (− 19 to 28)	0.575
ED	7 ± 7 (− 3 to 19)	6 ± 9 (− 14 to 22)	0.775
ES-ED *p* value	0.233	0.942	

The directional positions of the SVC were observed to strongly correlate with RA volume. The RA volume exhibited strong correlations with the basal and anterior SVC distances, with no correlation in the lateral direction (Right Column of Figure 7). Correlation was additionally utilized to assess any relationships between each of the SVC positional directions. The basal and anterior distances exhibited a moderate degree of correlation (*r* = 0.57, 95% CI 0.41–0.69, *p* < 0.0005) whereas weak to no correlation was observed between the other directional components (basal vs. lateral directions: *r* = − 0.05, 95% CI − 0.25 to 0.16, *p* = 0.630; anterior vs. lateral directions: *r* = 0.06, 95% CI − 0.15 to 0.26, *p* = 0.587).

### Annulus Excursion

Directional excursions of the tricuspid annulus centroid from end-diastole to end-systole were determined. Since transcatheter systems are known to approach the right heart from the IVC and SVC, the excursion was measured relative to both of the caval centroids (Table 6). Overall, the total displacement of the annulus centroid was similar between and within groups. This displacement was primarily in the basal-to-apical direction. Interestingly, the directional displacements of the annulus were observed to differ when measured from the IVC and SVC; however, the magnitude of these differences are unlikely to be practically meaningful.

**Table 6:** Directional excursion of the tricuspid annular centroid from end-diastole to end-systole measured relative to the inferior vena cava (IVC) and superior vena cava (SVC) expressed as a mean ± SD (minimum-maximum), where ζ, * , *γ* , and *ψ* denote statistically significant differences in the total, basal, anterior, and septal excursions within the ≤ 1 + TR and ≥ 3 + TR groups at the *p* < 0.05 level.

Excursion	≤ 1 + TR	≥ 3 + TR	*p* value
Relative to the IVC (mm)			
Total	15 ± 7 (2–28)	12 ± 5 (4–27)	0.064
Apical-to-basal	11 ± 6 (− 1 to 23)	10 ± 5 (2–22)	0.385
Posterior-to-anterior	1 ± 5 (− 9 to 11)	3 ± 5 (− 6 to 15)	0.296
Lateral-to-septal	6 ± 6 (− 3 to 16)	4 ± 4 (− 6 to 13)	0.155
Relative to the SVC (mm)			
Total	11 ± 3 (7–16)ζ	9 ± 4 (3–21)ζ	0.020
Apical-to-basal	6 ± 5 (− 2 to 16)*	6 ± 4 (− 1 to 19)*	0.794
Posterior-to-anterior	7 ± 3 (2–12)^γ^	3 ± 4 (− 6 to 15)^γ^	0.003
Lateral-to-septal	2 ± 5 (− 6 to 10)^ψ^	0 ± 4 (− 7 to 9)^ψ^	0.280

### Inter-Software and User Variability

**Figure 8:**
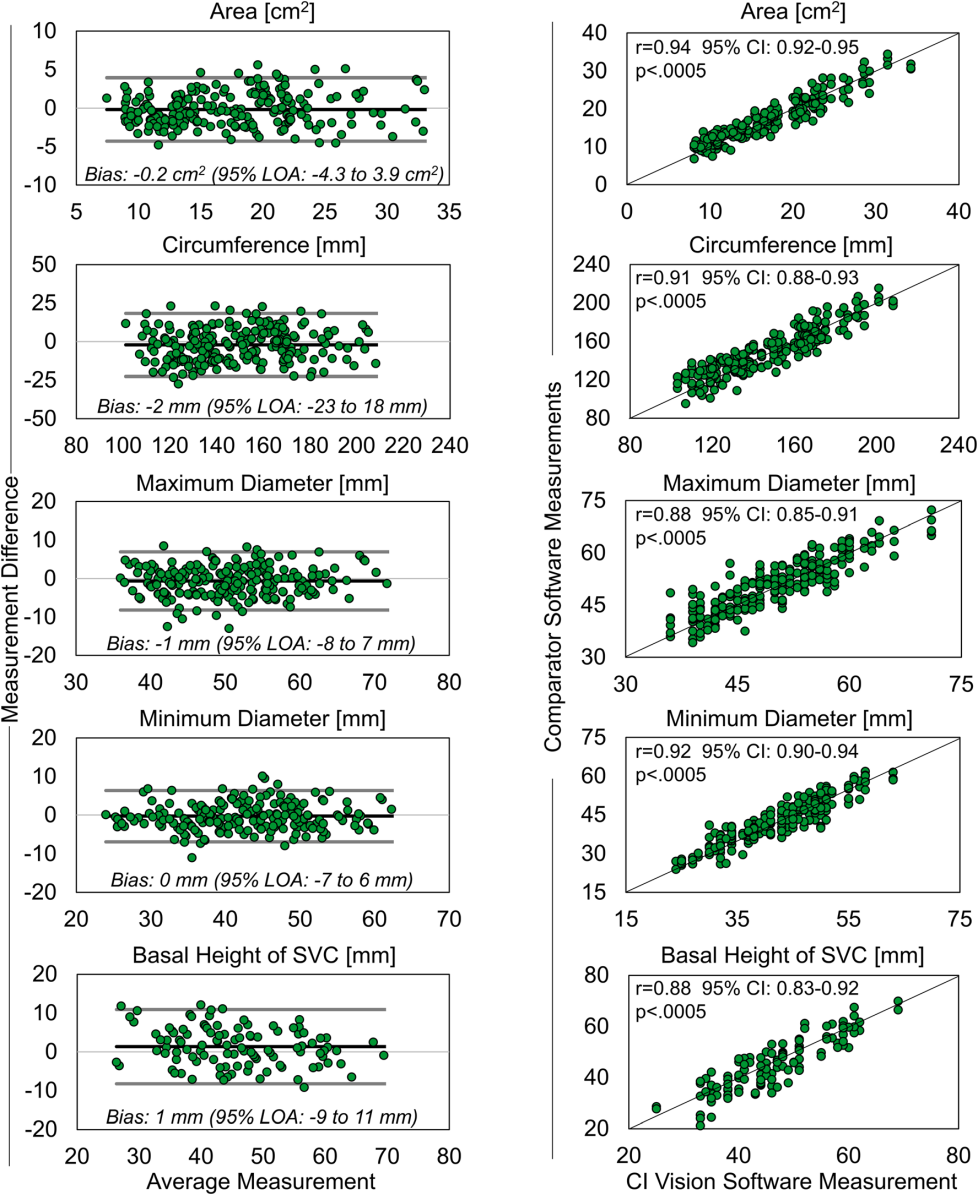
Intra-software variability results where the left column contains Bland-Altman plots for the evaluated measures with mean bias and 95% limits of agreement (LOA) shown; right column contains the corresponding correlations between the CI vision-based measurements (*x* axis) and the comparator software measurements (*y* axis). Note: basal height measurements of the SVC using 3Mensio was completed using a definition differing from Figure 2, and therefore was omitted from the variability analysis.

The precision and agreement of measurements by CI Vision to Mimics, 3 Mensio, and HeartNavigator are presented in Figure 8. The basal height measurements of the SVC using 3Mensio were completed using a definition differing that of Figure 2, and therefore were omitted from the variability analysis. All measurements were observed to exhibit low levels of precision bias with good and acceptable limits of agreement given the differing capabilities in heart segmentation, workflow functionalities, availability of feature identification, and degrees of automation within the tested comparator software. Measurements between the comparator software and CI Vision were strongly correlated and found to be in good agreement.

## Discussion

This study quantitatively evaluated the phasic right heart morphology of transcatheter patient candidates with moderate or greater FTR and of subjects with trace or no regurgitation. These comprehensive data significantly supplement and bridge critical gaps in right heart morphology knowledge and data. In their totality, these data provide an initial platform from which the variable right heart morphologies in these patients can continue to be better understood for further improving transcatheter therapies.

### Studied Patient Groups

CCTA is frequently performed in transcatheter TV patient screening for its excellent temporal and spatial resolution. This modality however not indicated for use in healthy persons due to is controlled use of ionizing radiation. Thus, in order to evaluate the impact of right heart disease on the newly reported right heart measures, the ≤ 1 + TR group consisted of patients with trace to no TR who underwent a CCTA for a transcatheter aortic valve replacement. While the ≤ 1 + TR group is not free of heart disease, the echocardiography characteristics, RA volumes, and RV volumes of this group were comparable to values reported in healthy persons from historic literature.^1,14,25^

The ≥ 3 + TR group exhibited a wide spectrum of transcatheter TV patient candidates. The selected patients were inclusive of patients which may be treated with edge-to-edge repair devices, TV replacement valves, leaflet spacers, annuloplasty systems, and heterotopic caval valve implantations. There were also patients selected for this study who were in more severe stages of the disease, providing excellent data for informing the potential extremes of the morphological measures. The resulting data are therefore believed to provide a reasonable assessment for the potential range and variability of morphological features that exist within the transcatheter TV population that can be supplemented and further improved with future studies.

### The Vena Cava

Nearly all transcatheter TV therapies traverse the vena cava but there is few available data quantifying their orientations and positions relative to the TV.^3^ The phasic vena cava positions and delivery axes were reported for the first time in this study. The delivery axis angles exhibited relatively small variations from end-diastole to end-systole. The IVC axis was observed to enter the atrium at a more parallel orientation to the tricuspid annular plane whereas the SVC entered the atrium angled more towards the annular plane. The delivery axis heights above the annulus were markedly increased within the ≥ 3 + TR group, as were the positional distances of the vena cava with respect to the annulus center.

Patient RA volumes were found to be the strongest predictor of vena cava position. In the slow progression of FTR, the RA enlarges, displacing the positions of the vena cava relative to the TV. For the IVC in particular, this displacement is primarily in the basal and to a lesser extent the posterior direction. For the SVC, the rate of distention is similar in both the basal and anterior directions (Figure 7).

For both vena cava, no readily observable trends were observed for their septal and lateral positions, respectively. The wide variations of the IVC and SVC position in the septal-lateral dimension is cause for attention, as positions in either direction exhibit potential to influence delivery system design, as well as potentially impact delivery system maneuvering, positioning, and device delivery.

Quantifying the size and directional position of the vena cava provides a framework for delivery system development, evaluation, and for planning delivery system positioning toward the annulus center (transcatheter valve replacement), or towards various points within or around the annular perimeter (edge-to-edge repair, annuloplasty, chamber reducing devices). For planning a delivery system’s approach, it is important to consider operator technique and the differing ways and routes by which the system may be positioned to the target location. Access site location and restrictions imposed on the device by the vasculature may also influence a device’s course, and therefore several strategies for achieving a given position may wished to be considered. Knowledge in the maneuverability of the device’s delivery system helps in this regard and is beginning to be included within select device’s instructions for use. Importantly, the multiple phases of the CCTA scan provide an ability to predict the dynamic range occurring during the procedure, further aiding this evaluation.

### Annular Tissue Shelf

Circumferential quantification of the annulus tissue shelf morphology was reported for the first time in this study. The mean results from the ≥ 3 + TR group exhibited a relatively unchanging shelf from the anterior to posterior region, a shallowed shelf near the coronary sinus, and a shelf verticalization at the aortic continuity. Select similarity in the tissue shelf was observed between the studied groups, with ≥ 3 + TR being most associated with a shallowing of the posteroseptal aspect of the shelf at or near the coronary sinus. Assessing the potential range in shelf lengths and angles was limited by the fully automatic quantification of the shelf measurements based on a 5-mm atrial height to the annular plane (Figure 2). While limiting, a 5-mm atrial height was selected anecdotally, as devices aim to assert their affects near the leaflet hinge, to maximize their effect on leaflet closure or to replace valve function.

Quantifying the shelf morphology can inform device design, delivery system development and testing, and procedural development. TV replacement valves for example must expand into the annular perimeter, secure themselves partially or fully to the annular shelf tissue, and seal off the annular shelf space to prevent perivalvular leakage. For repair and annuloplasty devices, the tissue shelf morphology helps by defining the appropriate delivery system articulations that would be required to achieve a desired anchor, suture, or attachment orientation to or within the shelf tissue. A shorter or vertically oriented shelf may be a challenge for anchoring systems which require a perpendicular angle of insertion from either the atrium or the ventricle. The ability to achieve these required angles of insertions are undoubtably influenced by the vena cava positions as well as delivery system maneuverability as previously described.

### Annulus Dimensions

The annulus dimensions and clinical characteristics of the ≥ 3 + TR group were consistent with ≥ 3 + TR and prior quantitative studies.^1,7,8,18,21^ A wide range of annular dimensions were observed in this study with overlap between the studied groups (Table 3, Figure 4). In considering these data along with the echocardiography-based measures of regurgitation and leaflet geometry, this overlap represents the spectrum of annulus geometries for which greater than moderate TR can occur, and similarly the range for which trace to no TR can exist. These data reflect the complexity of mechanisms which are known to lead to TR, which include not only annular enlargement, but also variable degrees of leaflet tethering and atrial (atriogenic) enlargement.

These CCTA based results support and supplement the echocardiography-based results of Mararu et al.^18^ in demonstrating the RA volume to strongly correlate with annular dimensions, and to a lesser degree, the RV volume. Historically, FTR is referenced as a ventricular disease; however, as emphasized in these collective works, pathological combinations of RA-RV volumes exist within the FTR population, whose implications for device design, device-patient selection, and treatment are for further discussion.

### Annulus to RCA Distance

Quantifying annulus-to-RCA distance is performed during patient screening for therapies in which the annular shelf is penetrated by the implant or its attachments.^6,8,13,21,27^ No practical differences in annulus-to-RCA distances were observed between the studied patient groups. This finding is consistent with prior studies showing no difference in annulus-to-RCA distance between FTR patients with TR ≥ 3 + and those with TR < 3 +, and the annulus-to-RCA distance being independent of sex, age, body weight, and etiology.^13,27^

The bi-phasic measurements of annulus-to-RCA distances support recent work by Hinzpeter et al. who demonstrated less than ± 1 mm of annulus-to-RCA distance changes through ten phases of the cardiac cycle.^8^ In absence of practically meaningful cyclic variations, these data suggest annulus-to-RCA distance measurements could be accomplished in a single cardiac phase for varying device or procedural planning purposes.

## Limitations

The study was retrospective. The study sample size is small relative to clinical trials but exceeds or is comparable in size to studies evaluating right heart anatomical features in patients with moderate or greater FTR (sample size range: *N* = 8–40).^8,15,16,20,21,27^ The sample size of this study is however lesser in size than a control arm of one study, in which the annulus-to-RCA distances and IVC dimensions were quantified in subjects with < 3 + TR (*N* = 210).^27^ In a subset of patients, the annulus-to-RCA distance was unable to be determined in the posterolateral and posterior segments due to the RCA diameter decreasing to or below the spatial resolution of the CCTA images, insufficient intraluminal contrast, or both. Some measures used in select transcatheter therapies were not quantified, including IVC-hepatic vein measures, TV-to-RV measurements, and outflow tract geometry. Interested readers may consider using the RV measurements taken by echocardiography to supplement the CCTA data, as applicable (Table 1**)**. The tricuspid leaflets were not analyzed using CCTA, although this is possible in some cases and is improving with technology. Echocardiography remains the primary modality for this purpose.^5^

## Conclusions

This study provides new and further insight to the right heart morphology and functional characteristics of patients with FTR. These preliminary insights underline the increasingly recognized variability and complexity of the right heart, and particularly some of the requirements for which transcatheter procedures must successfully operate within and treat. In their totality, these data provide an initial platform from which the variable right heart morphologies in these patients can continue to be better understood for further improving transcatheter system design, sizing, and device-procedure development.

## Supplementary Information

Below is the link to the electronic supplementary material.

Supplementary file1 (DOCX 20 kb).
