# Supplementary material for: Right Heart Morphology of Candidate Patients for Transcatheter Tricuspid Valve Interventions
Source: Cardiovasc Eng Technol. 2021 Dec 1;13(4):573–89. doi: 10.1007/s13239-021-00595-y (PMC9499909; doi:10.1007/s13239-021-00595-y)
Supplement: Supplementary file 1 — Supplementary file1 (DOCX 20 kb). [file 13239_2021_595_MOESM1_ESM.docx]

Supplemental Table 1. Geometry of the inferior vena cava opening to the right atrium.

| Measurement | ≤1+TR | ≥3+TR | p-value |
| --- | --- | --- | --- |
| Maximum Diameter [mm] |  |  |  |
| ES | 28±5 (18-38) | 34±6 (25-45) | 0.002 |
| ED | 29±4 (22-38) | 33±6 (25-48) | 0.006 |
| p-value | 0.869 | 0.206 |  |
| Minimum Diameter [mm] |  |  |  |
| ES | 24±4 (17-31) | 28±4 (20-34) | 0.021 |
| ED | 26±4 (19-32) | 27±4 (17-38) | 0.290 |
| p-value | 0.095 | 0.674 |  |
| Area [cm^2^] | |  |  |
| ES | 5.5±1.7 (2.4-9.5) | 7.5±2.0 (4.1-11.5) | 0.002 |
| ED | 5.9±1.8 (3.7-9.5) | 7.2±2.2 (3.3-14.3) | 0.040 |
| p-value | 0.240 | 0.332 |  |
| Perimeter [mm] | |  |  |
| ES | 83±13 (68-110) | 97±14 (73-122) | 0.003 |
| ED | 86±13 (18-38) | 95±15 (66-135) | 0.029 |
| p-value | 0.289 | 0.263 |  |

Supplemental Table 2. Geometry of the superior vena cava opening to the right atrium.

| Measurement | ≤1+TR | ≥3+TR | p-value |
| --- | --- | --- | --- |
| Maximum Diameter [mm] |  |  |  |
| ES | 20±3 (15-27) | 29±4 (22-39) | <0.0005 |
| ED | 18±3 (13-24) | 29±5 (21-39) | <0.0005 |
| p-value | <0.0005 | 0.185 |  |
| Minimum Diameter [mm] |  |  |  |
| ES | 17±3 (12-23) | 26±5 (18-36) | <0.0005 |
| ED | 15±4 (11-24) | 24±4 (17-34) | <0.0005 |
| p-value | 0.104 | 0.002 |  |
| Area [cm^2^] | |  |  |
| ES | 2.7±1.0 (1.7-4.8) | 6.0±1.9 (3.3-10.3) | <0.0005 |
| ED | 2.2±0.9 (1.2-4.6) | 5.6±1.8 (3.1-10.2) | <0.0005 |
| p-value | 0.001 | 0.004 |  |
| Perimeter [mm] | |  |  |
| ES | 58±10 (46-79) | 86±14 (64-114) | <0.0005 |
| ED | 52±10 (39-76) | 84±13 (63-114) | <0.0005 |
| p-value | <0.0005 | 0.008 |  |
